# Seasonal Variation of Microplastic Contamination in Landfill Leachate, River Water, and Soil During Premonsoon and Monsoon: First Evidence From Landfill Sites in Nepal

**DOI:** 10.1155/jt/5660004

**Published:** 2026-08-03

**Authors:** Yubraj Dahal, Saroj Babu Koirala, Ramesh Gautam, Rashika Pandit, Reema Lama Pakhrin, Rabindra Prasad Dhakal, Sandhya Babel

**Affiliations:** ^1^ Faculty of Technology, Nepal Academy of Science and Technology, Lalitpur, Nepal, nast.gov.np; ^2^ School of Bio-Chemical Engineering and Technology, Sirindhorn International Institute of Technology, Thammasat University, Pathum Thani, Thailand, tu.ac.th

**Keywords:** agriculture, leachate, microplastics, monsoon, soil

## Abstract

Landfills serve as a significant source of microplastics (MPs) contamination, affecting the surrounding air, water, and agricultural soils. This study examined the seasonal variation of MPs in leachate, river water, and soil at Sisdole (old) and Banchare Dada (new) landfill sites (LFSs). MPs greater than 90 μm were enumerated using a stereomicroscope, and polymer identification was performed on a representative subset of particles using micro‐Fourier transform infrared (FTIR) spectroscopy. Average MPs concentrations were higher at the new landfill, with values of 56 ± 07 MPs/L at Banchare Field and 44 ± 27 MPs/L at Banchare Tank, compared to 28 ± 11 MPs/L at Sisdole Pond in the old landfill. In river water, MPs increased downstream: 17 ± 12 MPs/L at Sisdole Upstream, 28 ± 22 MPs/L at Sisdole Downstream, and 34 ± 06 MPs/L at Banchare Downstream. Soil contamination was highest at Sisdole (351 ± 173 MPs/100 g), followed by Banchare (214 ± 70 MPs/100 g) and Banchare‐Sisdole (157 ± 48 MPs/100 g). Statistical analyses, including a paired *t*‐test for river water and a two‐way ANOVA for soil samples, showed significant differences in MPs concentrations between the monsoon and premonsoon seasons in both river water and soil. Fibers were the dominant form of MPs in leachate, river water, and soil, whereas fragments were mainly within the 90–500 μm size range and fibers within the 1000–5000 μm range. Polyethylene terephthalate/polyester (PET/PES) accounted for 50% of the identified polymers, followed by polypropylene (PP, 20%), polyethylene (PE, 10%), and other polymers (20%). The polymeric composition suggests that textiles and degraded plastic packaging are the possible sources of MPs. Overall, the study identifies landfill‐derived MPs as a possible source of pollution in nearby river and agricultural lands, underscoring the urgent need for measures, such as leachate treatment and improved landfill management practices by the relevant authorities.

## 1. Introduction

Plastics are synthetic materials that have become one of the most common forms of waste and a major environmental concern. Global plastics production rose from 370.6 million tons (Mt) in 2018 to 430.9 Mt in 2024, with fossil‐based plastics dominating and circular‐based plastics contributing only a small fraction [1]. Over time, these plastics break down into smaller fragments, forming MPs and even smaller nanoplastics (NPs).

MPs are tiny plastic materials, including fibers, particles, or films, measuring less than 5 mm in length [2, 3]. They are typically classified into two categories: primary, which are deliberately manufactured small plastics, and secondary, which result from the breakdown of larger plastic items [4]. Primary MPs are intentionally produced for specific uses. They are manufactured for use in various consumer and commercial products, such as cosmetics, cleaning agents, paints, pharmaceuticals, diapers, and insecticides [5]. These particles can escape into the environment during production, transport, or regular use. In contrast, secondary MPs can also form unintentionally from the breakdown of larger plastics due to physical, chemical, or biological processes, such as the degradation of tire debris, plastic bags and bottles, food containers, and fishing gear [6, 7]. Although MPs are less visible, they can pose significant environmental risks [8]. Due to their widespread distribution and persistence, MPs have emerged as a global environmental and health concern [9].

While early research primarily focused on aquatic systems, MPs have increasingly been detected in terrestrial environments, including soils and landfill leachate [10, 11]. It is estimated that 21%–42% of global plastic production ultimately ends up in landfills, with the main source being solid waste and residual materials from wastewater treatment, such as sludge and fats, oils, and grease [8, 12]. Moreover, the buried plastics in landfills are exposed to harsh conditions, including fluctuating leachate pH (4.5–9), high salinity, variable temperatures, gas generation (e.g., CO_2_ and CH_4_), mechanical stress, and microbial degradation, which accelerate their fragmentation into MPs [13]. Poor waste management and the gradual fragmentation of plastics facilitate MPs moving into deeper soil and nearby water, making landfills a continuous source of contamination [14]. The infiltration of rainwater promotes the transport of MPs into leachate, thereby enabling their migration to adjacent sediments and groundwater via leaching and infiltration processes, especially in inadequately designed landfills [15]. Moreover, MPs may also be introduced into agricultural systems via leachate‐contaminated irrigation water and atmospheric deposition. Thus, landfills are emerging sources of MPs, affecting surrounding land and water ecosystems.

In aquatic systems like rivers, MPs can be ingested by a wide range of organisms, potentially leading to the bioaccumulation and biomagnification of associated contaminants through the food chain [16]. Moreover, MPs ingestion by aquatic organisms may result in various adverse effects, including disruptions in reproduction and growth, oxidative stress, reduced survival, and alterations in gut microbiota [17]. Once in the soil, MPs alter physicochemical properties and reshape microbial communities, thereby affecting the cycling of carbon, nitrogen, and phosphorus [18]. The extent of these impacts depends on MPs’ characteristics, such as type, size, concentration, polymer composition, surface properties, and exposure duration [19]. In subsoils, MPs can disrupt microbial diversity and interactions, further intensifying nitrogen limitation [20, 21].

Furthermore, studies report that MPs can influence soil carbon dynamics by increasing soil carbon content, microbial biomass, and CO_2_ emissions, while also altering nitrogen transformations by reducing nitrate and ammonia losses [22]. In addition, MPs contamination may affect microbial carbon use efficiency, with soil nutrient status interacting with MPs to regulate carbon cycling in soil ecosystems [23]. Beyond soil processes, MPs can also impact plant growth and development through uptake and translocation within plant tissues [24], where they may induce oxidative stress, damage root cells, disrupt enzyme activity, and reduce photosynthetic efficiency and chlorophyll content [25]. Overall, MPs pose significant risks to soil health and agricultural productivity.

The concentration of MPs in leachate varies across the studies. The highest concentration recorded so far is 4010–33213 MPs/L in a landfill in Guangzhou, China [26]. Similarly, the lowest is 1.2 ± 0.57 MPs/L in Shanghai, China [27]. Landfill leachate is dominated by both fibers and fragments [28–31]. Fibers or lines are elongated, thin MPs with one dimension longer than the others, whereas fragments are thicker particles with irregular shapes [32]. The criteria for identifying plastic particles or fibers are as follows: (1) they should not exhibit any cellular or organic structures; (2) if the particle is a fibers, it must maintain a uniform thickness, not taper at the ends, and display three‐dimensional bending, as straight fibers often indicate a biological origin; (3) the particles should be clear and uniformly colored, typically blue, red, black, or yellow; and (4) if the particle appears transparent or whitish, it should be examined carefully under high magnification and fluorescence microscopy to confirm it is not of organic origin [33]. Regarding polymer type, PP, PE, and PET are the most common polymers [26, 28, 34, 35]. Similarly, the concentration of MPs reported in soil samples varies from 66 MPs/kg (Bangladesh) to 60111.67 MPs/kg (Indonesia) [28, 36]. Similar to the leachate, fibers and fragments are the most common shapes of MPs, and PP and PE are frequently observed polymers in soil samples [14, 30, 36, 37]. The similarity in MPs’ shape and polymer composition between leachate and soil samples suggests that landfills act as sources of MPs. In contrast, surrounding soils serve as sinks, with MPs potentially migrating beyond the landfill boundary.

Due to their persistence and widespread distribution, MPs have emerged as a global environmental threat, raising serious concerns about their potential risks to human and ecosystem health. Understanding their occurrence, sources, transport pathways, and impacts is essential for mitigating their long‐term ecological and health effects. Despite this, research on MPs contamination in landfill leachate, soil, and leachate‐contaminated water is unexplored in Nepal. To fill this knowledge gap, leachate, soil, and river water samples were collected from different locations at the Sisdole and Banchare Dada LFSs during the premonsoon and monsoon seasons of 2024. Leachate samples were collected from a natural collection pond, an active waste disposal area, and a designated leachate collection tank, while river water samples were collected from upstream and downstream sections of the Sisdole and Banchare Dada LFSs. Soil samples were collected at depths of 0.25, 0.50, and 0.75 m from leachate‐impacted areas and adjacent agricultural lands. This study analyzed and compared MPs concentrations across leachate, river water, and soil, identified potential factors influencing spatial and seasonal variations, and provided recommendations for regulatory authorities and future research.

## 2. Methodology

### 2.1. Study Area

**FIGURE 1:**
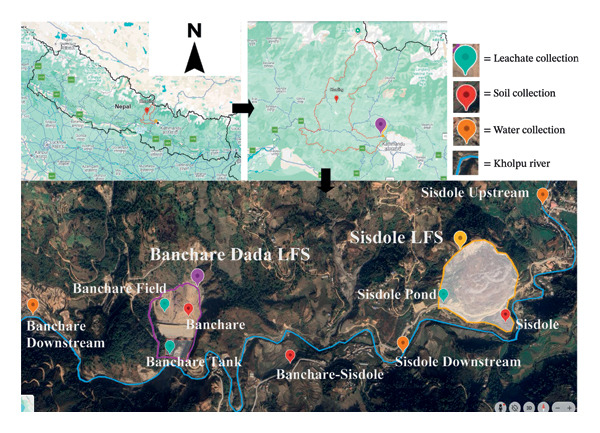
Location of the sample collection sites.

Samples were collected from Banchare Dada and Sisdole LFSs (Figure 1). The Sisdole LFS is located in Okharpauwa, Kakani Rural Municipality, Nuwakot District, about 25 km northwest of Kathmandu, on the northern bank of the Kolpu River at an elevation of approximately 1150 m above sea level. It served as the primary dumping site for municipal solid waste (MSW) from the Kathmandu Valley between 2005 and 2022 until its closure, with daily waste intake increasing from approximately 155 tons per day in 2005 to 830 tons per day in 2021 [38]. The LFS spans 15 ha and was originally intended as a temporary waste disposal site with a capacity of just 2 years.

After the closure of the Sisdole LFS, the Banchare Dada LFS has been in operation since 2022, located approximately 2 km away from Sisdole, as a more sustainable and long‐term solution. Currently, the landfill receives an estimated daily waste input of around 1000 tons [38]. Covering an area of 23 ha, Banchare Dada has been designed with basic landfill infrastructure, such as lined cells to minimize soil contamination and provision for leachate collection. However, it lacks advanced management facilities, such as leachate treatment systems, and the leachate is discharged into the downstream Kholpu River.

### 2.2. Sample Collection

Leachate, river water, and soil samples were collected from the study sites during two sampling campaigns conducted in the premonsoon (April 2024) and monsoon (July 2024) seasons. At the Sisdole LFS, leachate was sampled from a natural collection pond (Sisdole Pond) located in the southern part of the LFS, which receives limited leachate discharge. During the monsoon, this pond was observed to overflow, releasing leachate into the Kholpu River. At the Banchare Dada LFS, leachate samples were collected from both the active waste disposal area (Banchare Field) and the designated leachate collection tank (Banchare Tank). River water samples were collected from three locations along the Kholpu River: upstream near Sisdole (Sisdole Upstream), downstream near Sisdole (Sisdole Downstream), and downstream near Banchare (Banchare Downstream). It was also observed that untreated leachate from the Banchare Tank was directly discharged into the Kholpu River. At each sampling point, triplicate 1 L samples were collected from slightly different spots within the site during both sampling seasons. Two replicates were used for MPs quantification and statistical analysis, while the third replicate was used for polymer identification and was therefore not included in the quantitative analysis.

Soil samples were collected at depths of 0.25, 0.50, and 0.75 m at each sampling point as individual cores and analyzed separately. Leachate‐affected soils were obtained from Sisdole LFS (Sisdole) and the Banchare Dada LFS (Banchare), along with additional samples from agricultural land located between the two sites (Banchare‐Sisdole). At each location, approximately 500 g to 1 kg of soil was collected from each depth. In the laboratory, samples were air‐dried at room temperature, and 5 g subsamples were analyzed in duplicate for MPs enumeration. The geographical coordinates of the sampling sites are provided in Table 1.

**TABLE 1:** Geographical locations of the sampling sites.

Sample type	Site code	Geographical coordinates
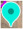 Leachate	Sisdole Pond	27°46′33.93″N 85°14′34.54″E
	Banchare Field	27°46′32.36″N 85°13′41.99″E
	Banchare Tank	27°46′25.73″N 85°13′42.96″E

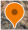 River water	Sisdole Upstream	27°46′48.62″N 85°14′52.39″E
	Sisdole Downstream	27°46′26.14″N 85°14′27.25″E
	Banchare Downstream	27°46′31.87″N 85°13′15.87″E

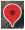 Soil	Sisdole	27°46′30.87″N 85°14′45.86″E
	Banchare‐Sisdole	27°46′24.54″N 85°14′06.06″E
	Banchare	27°46′31.66″N 85°13′46.67″E

### 2.3. Sample Treatment and Enumeration

The leachate samples were first sieved using a 90 μm mesh to separate larger particles. The retained fraction was thoroughly rinsed with distilled water and transferred to a 250 mL beaker. The samples were then oven‐dried at 50°C to enhance digestion efficiency. After drying, they were treated with 20 mL of 30% hydrogen peroxide and 0.50 mL of Fe (II), following the procedure described by Dahal and Babel [39]. The mixture was stirred at 200 rpm to ensure uniform mixing and accelerate digestion. Digestion was allowed to proceed until bubbling ceased and the solution became clear. Subsequently, the samples were heated to 45°C to decompose residual hydrogen peroxide into water and oxygen, ensuring complete digestion while avoiding re‐sieving, which could result in the loss of MPs.

Following digestion, approximately 100 mL of zinc chloride solution (density 1.5 g/cm^3^) was added for density separation, as most plastics have a lower density than this value. The mixture was left undisturbed for 24 h, after which the supernatant was vacuum‐filtered through a 0.45 μm cellulose nitrate membrane filter. The filters were then air‐dried at room temperature and examined under a stereomicroscope (SZ2‐ILST, Olympus Corporation, Tokyo, Japan) for sorting and counting. MPs were classified into fibers and fragments following the method described by Dahal and Babel [39]. The color of each particle was also recorded during microscopic observation. The size of the selected fibers and fragments was measured using the open‐source ImageJ software [40]. MPs were sorted into three different ranges: 90–500, 500–1000, and 1000–5000 μm.

A 5 g portion of each soil sample was placed in a 250 mL beaker, followed by the addition of 30 mL of 30% hydrogen peroxide and 0.50 mL of Fe (II). The mixture was stirred at 200 rpm to ensure thorough mixing and accelerate digestion. After digestion, the soil samples were further processed using the same protocol applied to the leachate samples.

### 2.4. Polymeric Analysis

Four bulk leachate samples were prepared for polymer identification. The premonsoon and monsoon samples collected from Sisdole Pond, Banchare Tank, and Banchare Field were combined for each site to form three bulk samples. Likewise, river water samples from both seasons were merged to form a single bulk sample. Soil samples were not analyzed separately for polymer composition, as they were collected from areas potentially influenced by leachate contamination and atmospheric deposition of landfill‐derived MPs. Therefore, the MPs detected in soil were assumed to primarily originate from landfill‐related inputs, with leachate‐derived MPs considered representative of the landfill source. Accordingly, the polymer composition of soil samples was assumed to be consistent with that identified from the bulk analysis.

From each filter/sample, 10 particles were analyzed using micro‐FTIR (Nicolet iN10, Thermo Scientific, USA) operated with Thermo Scientific OMNIC Picta software. The particles were randomly selected to provide a representative assessment of polymer composition, consistent with previous studies [39, 40] and recommended by Dahal and Babel [41], thereby capturing the dominant polymer types despite the limited subsample size. The instrument, equipped with a cooled MCT‐A detector for enhanced sensitivity, recorded each spectrum over 128 scans in 25 s at a resolution of 8 cm^−1^, covering the spectral range of 500–4000 cm^−1^. An adjustable aperture (50–250 μm in height and width) enabled microscopic targeting of individual particles. Ten particles (fibers and fragments) per filter were selected in proportions corresponding to those observed during stereomicroscopic examination of duplicate samples used for sorting and enumeration. This proportional approach was applied to adjust MPs counts after blank correction, minimizing potential over‐ or underestimation during stereomicroscopic analysis. A match factor threshold of 70% was used to confirm polymer identity [39, 41].

### 2.5. Background Contamination Prevention

To minimize background contamination, the use of plastic materials was avoided wherever possible. Six procedural blanks for each sample type (leachate and soil) were processed using the same chemicals and procedures as the actual samples, including exposure to laboratory air and identical handling at each step. This ensured that any airborne MPs contamination introduced during analysis could be accounted for. The measured background contamination was subtracted from the sample counts to obtain more accurate estimations.

### 2.6. Statistical Analysis

The results were organized and analyzed in Microsoft Excel, and graphs were prepared to enhance visualization. MPs concentrations at each sampling point are reported as mean ± SD (*n* = 2 for leachate and river water; *n* = 6 for soil). Overall site averages across both seasons are reported as mean ± SD (*n* = 4 for leachate and river water; *n* = 12 for soil). Paired *t*‐tests were used to assess seasonal variation in MPs in landfill leachate and river water, while two‐way ANOVA was applied to evaluate the effects of season and soil depth on MPs abundance in soils.

## 3. Results and Discussion

### 3.1. MPs Count

Table 2 summarizes MPs concentrations for all sites, seasons, and matrices (leachate, river water, and soil)

**TABLE 2:** Concentration of MPs observed in leachate, river water, and soil during each sampling.

**Leachate**			

**Season**	**Sisdole Pond**	**Banchare Field**	**Banchare Tank**

Premonsoon (MPs/L)	19 ± 05	59 ± 11	26 ± 05
Monsoon (MPs/L)	36 ± 05	53 ± 01	62 ± 29

**River water**			

**Season**	**Sisdole Upstream**	**Sisdole Downstream**	**Banchare Downstream**

Premonsoon (MPs/L)	7 ± 08	10 ± 08	33 ± 08
Monsoon (MPs/L)	27 ± 02	46 ± 08	34 ± 06

**Soil**			

**Season**	**Sisdole**	**Banchare**	**Banchare-Sisdole**

Premonsoon (MPs/100 g)	386 ± 232	163 ± 63	123 ± 25
Monsoon (MPs/100 g)	315 ± 94	265 ± 22	191 ± 42

The mean concentration of MPs in leachate rose from 35 ± 20 MPs/L during the premonsoon period to 50 ± 18 MPs/L in the monsoon season; however, this variation was not statistically significant at *α* = 0.05 (*t*_5_ = −1.77, *p* = 0.137). Despite the lack of statistical significance, the trend indicates an increase in MPs loading in leachate during the monsoon period. Site‐wise, the average MPs concentration in leachate was 28 ± 11 MPs/L at Sisdole Pond, 56 ± 07 MPs/L at Banchare Field, and 44 ± 27  MPs/L at Banchare Tank.

In river water, the average MPs concentration increased from 17 ± 14 MPs/L in the premonsoon season to 36 ± 10 MPs/L during the monsoon, and this difference was statistically significant at *α* = 0.05 (*t*_5_ = −2.70, *p* = 0.043), indicating a marked rise in MPs levels in river water during the monsoon. The spatial distribution showed mean concentrations of 17 ± 12 MPs/L at Sisdole Upstream, 28 ± 22 MPs/L at Sisdole Downstream, and 34 ± 06 MPs/L at Banchare Downstream. For comparison, Table 3 summarizes the abundance of MPs in leachate reported in previous studies alongside the findings of the present study.

**TABLE 3:** Abundance and characteristics of MPs in leachate.

Country	Leachate type	Major MPs (μm)	Count (MPs/L)	Morphology	Polymers	Reference
Nepal	Raw	90–500	28–56	Fragments and fibers^∗^	PES/PET^∗^, PP, PE, and others	This study
Bangladesh	Raw, treated	500–1000	15–516	Fragments^∗^, films, fibers, pellets, and foams	PE^∗^, PP, PS, PVC, PET, PVA, PAN, PMMA	[28]
China	Raw	10–100	70–5320	Fragments^∗^, films, fibers, and pellets	PF^∗^, UF, PCTFE, POM^∗^, PS, and Others	[29]
Indonesia	Raw, treated	> 100	2100–4385	Fragments^∗^, films^∗^, fibers, foams, and pellets	HDPE, PP, and PA	[34]
China	Raw	20–100	4010–33213	Fragments^∗^ and fibers	PET^∗^, PU^∗^, PS^∗^, PP, PE, PVC, ACR, PMMA, and others	[26]
India	Raw	50–100	1473–2067	Fragments^∗^, film^∗^, fibers, microbeads, and foams	PET^∗^, PP, PVC, and CA	[35]
Sri Lanka	Raw	> 1000	0.76–4.48	Fragments^∗^, foams, fibers, beads, and lines	PE^∗^, PVC, PU, PA, CL, PP, PS, PEI, AC, PV, PET, PC, and ALK	[92]
Shenzhen, China	Raw	20–150	3–25	Fibers^∗^, films, and fragments	PU, PAT, PA, PEC, PP, PE, PS, PET, and others	[30]
China	Raw, treater	20–50	0.4–235.4	Fragments^∗^, fibers, films, and beads	PE^∗^, PVA, PVB, PP, PA, and others	[93]
Shanghai, China	Raw	< 500, 1000–5000	1.2 ± 0.57	Fibers^∗^, films, particles, and rods	PE^∗^, PES, PP, PA, EPM, and PVAC	[27]

Abbreviations: AC = Acrylate Copolymer, ACR = Acrylate Copolymer, ALK = Alkyd, CA = Cellulose Acetate, CL = Cellophane, EPM = Ethylene Propylene Polymer, HDPE = High Density Polyethylene, PA = Polyamide, PAN = Polyacrylonitrile, PC = Polycarbonate, PCTFE = Polychlorotrifluoroethylene, PEC = Polyethylene Carbonate, PEI = Polyetherimide, PF = Phenol Formaldehyde, PMMA = Polymethyl Methacrylate, PS = Polystyrene, PU = Polyurethane, PVAC/PVA = Polyvinyl Acetate, PVB = Polyvinyl Butyral, PVC = Polyvinyl Chloride, UF = Urea Formaldehyde Resin.

^∗^Dominant type of polymer or morphology.

**FIGURE 2:**
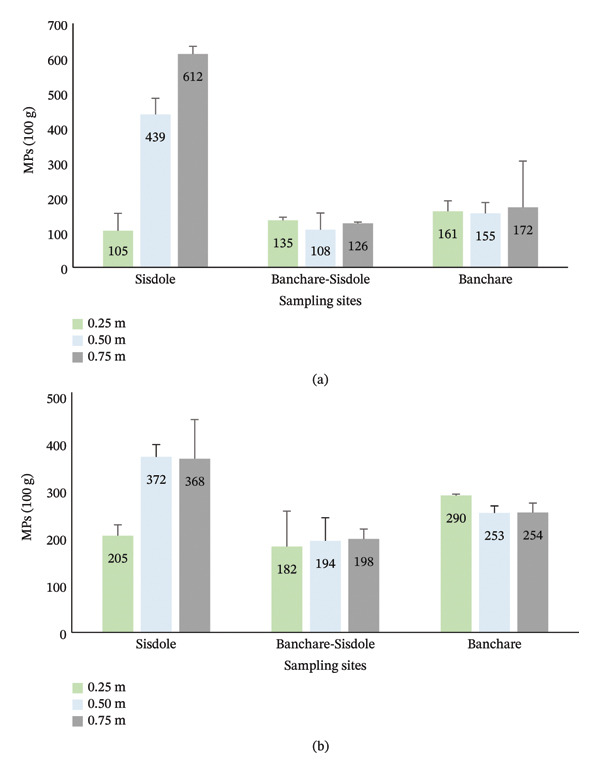
MPs observed in different soil depths during (a) premonsoon and (b) monsoon.

As illustrated in Figure 2, the distribution of MPs varied with soil depth in both the premonsoon and monsoon seasons. At Sisdole, MPs concentrations in soil decreased during the monsoon compared to the premonsoon period. Two‐way ANOVA revealed a significant seasonal effect at *α* = 0.05 (F_1,6_ = 6.76, *p* = 0.041). Soil depth also showed a highly significant influence (F_2,6_ = 54.91, *p* < 0.001), while a significant Season × Depth interaction (F_2,6_ = 13.42, *p* = 0.006) indicated that the reduction in MPs differed across soil layers.

At Banchare‐Sisdole, MPs concentrations in soil were slightly higher during the monsoon than in the premonsoon season. The seasonal effect was statistically significant (F_1,6_ = 7.64, *p* = 0.033), whereas soil depth had no significant effect (F_2,6_ = 0.07, *p* = 0.931). The Season × Depth interaction was also non‐significant (F_2,6_ = 0.21, *p* = 0.816), suggesting that seasonal variation occurred uniformly across all depths.

At Banchare, MPs concentrations increased during the monsoon relative to the premonsoon season. Two‐way ANOVA showed a significant seasonal effect (F_1,6_ = 9.43, *p* = 0.022), while soil depth had no measurable influence (F_2,6_ = 0.15, *p* = 0.868). The interaction between season and depth was likewise non‐significant (F_2,6_ = 0.16, *p* = 0.855), indicating a consistent increase in MPs across the soil profile.

Overall, the highest mean MPs concentration was observed at Sisdole (351 ± 173 MPs/100 g), followed by Banchare (214 ± 70 MPs/100 g), while the lowest concentration was recorded at Banchare‐Sisdole (157 ± 48 MPs/100 g). This pattern remained consistent across both seasons, with the overall trend: Sisdole (old) > Banchare (new) > Banchare‐Sisdole (agricultural land). For comparison, Table 4 presents the abundance of MPs in soil reported in previous studies alongside the findings of the present study.

**TABLE 4:** Abundance and characteristics of MPs in soil.

Country	Soil type	Major MPs (μm)	Count (MPs/kg)	Morphology	Polymers	Reference
Nepal	Landfill, agriculture	90–500	1570–3510	Fibers^∗^ and fragments	PES/PET^∗^, PP, PE, and others	This study
Bangladesh	Landfill	500–1000, 1000–5000	66–1510	Fragments^∗^, films, fibers, pellets, and foams	PE^∗^, PP. PS, PVC, PET, PVA, PAN, and PMMA	[28]
China	Landfill		592–47819	Fragments^∗^ and fibers	PET, PU, PS, PP, PE, PVC, ACR, PMMA, and others	[26]
Bangladesh	Agriculture	< 1000	293–1927	Films^∗^, fibers, fragments, and foams	PE^∗^, PP^∗^, PS, PET, and PAM	[64]
Indonesia	Landfill		60111.67	Fragments^∗^, films, fibers, and pellets	PE, PP, PVC, PS, PET, and PA	[36]
Iran	Landfill	100–500	225–863	Films^∗^, fibers, and fragments	LDPE^∗^, PET, PS, PP, PU, PVC, and PA	[49]
Republic of Korea	Agriculture	< 1000	73.4–97.8	Fragments^∗^, fibers^∗^, films^∗^, and spheres	PE^∗^, PP^∗^, PET, PS, PA, PMMA, and PVC	[37]
India	Landfill	600–1000	180–1120	Fragments^∗^, fibers, films, and foams	PE, PP, and EVA	[14]
China	Landfill	30–150	570–14200	Fibers^∗^, films, and fragments	PE^∗^, PP, PET, PS, PVC, and PU	[30]

Abbreviation: EVA = ethylene vinyl acetate.

^∗^Dominant type of polymer or morphology; agriculture = agricultural area near the LFS.

At Banchare Field, a large leachate pond had formed, accumulating a substantial volume of leachate generated from the active waste disposal area. In contrast, Sisdole Pond consisted of a relatively small natural leachate collection pond located downslope of the site (Figure 1). The leachate at Sisdole Pond originated from decomposing waste and percolated through multiple waste layers before collection. The comparatively lower MPs concentrations at Sisdole Pond, relative to Banchare Field, may be explained by the retention of MPs within these waste layers. Consistent with this study, Su et al. [42] reported a strong decrease in MPs abundance with landfill age, where younger landfill leachate (< 3 years) had much higher MPs concentrations (MPs/L) compared to older landfill leachate (> 20 years), which had only 4 MPs/L. Similarly, the lower MPs levels observed at Banchare Tank compared to Banchare Field could be due to the retention of MPs by both the daily soil cover applied after waste deposition and the waste mass itself. The elevated MPs concentrations during the monsoon season, compared to the premonsoon period, are likely associated with rainfall‐driven leaching, which enhances the mobilization of MPs from the waste mass. Consistent with this study, MPs concentrations in raw leachate were higher in autumn than in spring at two different leachate treatment plants (14.67 vs. 9 MPs/L and 14.67 vs. 6.5 MPs/L), indicating a consistent seasonal increase during autumn [43]. Similarly, Mohammadi et al. [44] reported an average MPs concentration of 79.16 MPs/L in leachate, with higher levels observed during summer and autumn. The seasonal variation was attributed to increased temperatures enhancing MPs abundance, while higher precipitation was associated with reduced MPs concentrations. Overall, these observations indicate that leachate collection characteristics, waste retention processes, and rainfall‐driven leaching collectively control the concentration and seasonal variability of MPs in landfill leachate systems.

As Sisdole Upstream is near the Sisdole LFS, the noticeable MPs concentration observed during the premonsoon period may be associated with atmospheric deposition of MPs potentially influenced by nearby landfill activities. Kannankai and Devipriya [45] suggested that atmospheric transport of MPs from landfills can act as a significant contamination pathway in nearby environments. This mechanism has also been widely documented in recent studies [46]. Similarly, landfill‐derived MPs have been identified on the surface of lichen samples collected near LFSs [47], indicating that MPs can disperse through the atmosphere and deposit in surrounding areas. Given that the Sisdole LFS lies adjacent to the Kholpu River, continuous atmospheric deposition may partly explain the higher MPs concentrations observed at Sisdole Downstream compared to Sisdole Upstream. In addition, leachate discharge is likely responsible for the elevated MPs concentration at Banchare Downstream during the premonsoon period. Collectively, these observations suggest that atmospheric deposition and leachate discharge from LFSs are key pathways influencing MPs distribution in the river system.

The marked increase in MPs concentration at Sisdole Upstream during the monsoon may be associated with surface runoff, which is suggested to mobilize MPs deposited on surrounding agricultural land into the river system. The further increase at Sisdole Downstream may be attributed to the transport of MPs from the Sisdole LFS via rainfall‐induced runoff. Moreover, a tributary joining the Kholpu River downstream of Sisdole Upstream may have contributed to increased river discharge during the monsoon, which could have led to reduced MPs concentrations at Banchare Downstream through dilution effects. Overall, these observations suggest that rainfall‐driven runoff, landfill proximity, and hydrological connectivity may influence the transport, redistribution, and dilution of MPs within the river system.

Sisdole LFS, which operated from 2005 to 2022, is considerably older than Banchare Dada LFS (operational since 2022). MPs in the leachate‐contaminated soils of Sisdole may have undergone prolonged environmental degradation and fragmentation, leading to higher concentrations compared to Banchare. This is consistent with landfill aging processes, where thermal degradation, biodegradation, and redox reactions during stabilization promote the generation and release of MPs [48]. As Banchare‐Sisdole is located between the two LFSs, MPs emitted from both landfills may have been deposited via atmospheric transport and subsequently incorporated into the soil through surface deposition and ploughing activities. Overall, these findings indicate that landfill age and proximity to multiple emission sources play a crucial role in controlling the accumulation and spatial distribution of MPs in surrounding soils.

At Sisdole, the observed decrease in MPs during the monsoon may be attributed to downward transport and leaching within the soil profile, along with surface erosion, as the site is located on a landfill slope that promotes both infiltration and soil loss. In contrast, Banchare‐Sisdole and Banchare exhibited increased MPs concentrations during the monsoon, likely due to rainfall‐driven runoff and hydrological transport processes that mobilize MPs from surrounding areas and facilitate their accumulation in soil. Though direct comparison is limited due to differences in soil depth, Shirazi et al. [49] reported that MPs abundance in shallow soil samples (0–3 cm) was similar in dry (863 ± 681 MPs/kg) and wet (861 ± 454 MPs/kg) seasons, while deep soil samples (3–6 cm) showed lower but more variable concentrations in dry (225 ± 138 MPs/kg) and wet (385 ± 198 MPs/kg) seasons. In contrast to the present study, which showed a site‐wise trend of Sisdole (old) > Banchare (new) > Banchare‐Sisdole (agricultural land), Shirazi et al. [49] reported that MPs levels were highest at active landfill areas and lower at closed and peripheral locations such as the MRF station. Overall, these patterns suggest that both seasonal rainfall and soil depth play significant roles in governing MPs distribution.

### 3.2. Morphology of MPs

Fibers dominated in both leachate and river water, accounting for 62% and 60%, respectively, while fragments comprised 38% and 40%, respectively. Among leachate samples, the highest proportion of fibers was observed at Banchare Field (68%), followed by Sisdole Pond (60%) and Banchare Tank (59%). In river water, fibers were most abundant at Sisdole Downstream (74%), followed by Banchare Downstream (59%) and Sisdole Upstream (49%).

**FIGURE 3:**
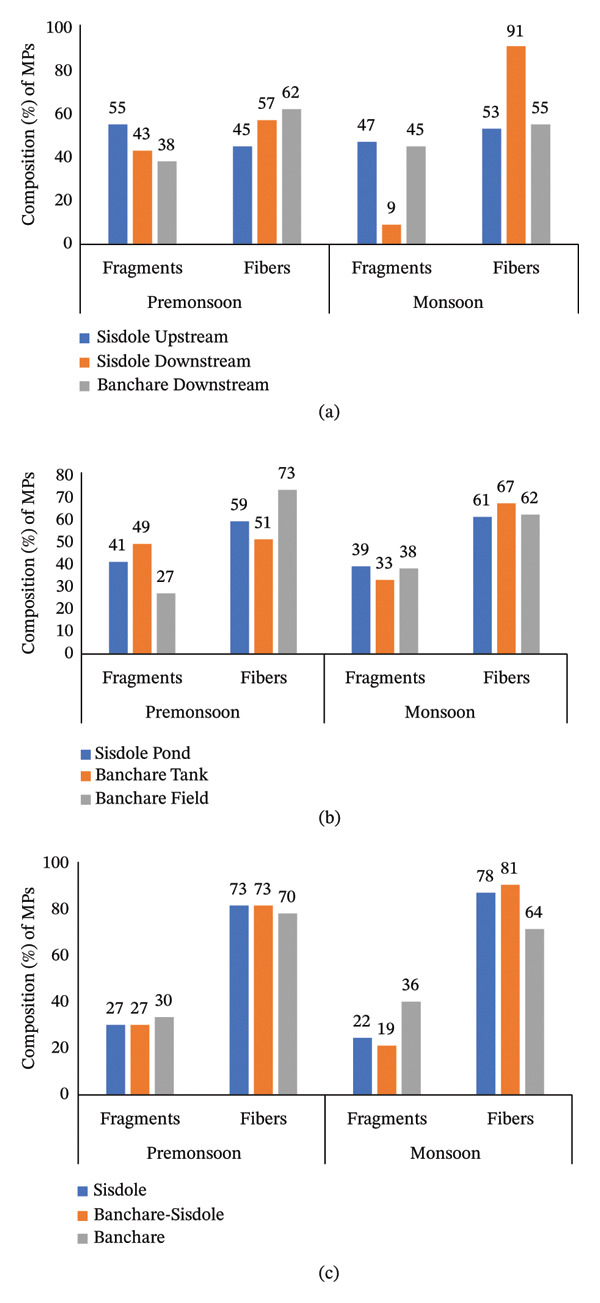
Composition of MPs in (a) river water, (b) leachate, and (c) soil.

Seasonally, fiber concentrations in leachate increased during the monsoon compared to the premonsoon period at Sisdole Pond and Banchare Tank, whereas a decrease was observed at Banchare Field. Similarly, fiber proportions increased at Sisdole Upstream and Sisdole Downstream (Figure 3), but decreased at Banchare Downstream. The morphology of MPs in leachate reported in previous studies is summarized in Table 3 for comparison with the present findings.

In soil, fibers accounted for 73% and fragments for 27%. Site‐specific composition showed 76% fibers and 24% fragments at Sisdole, 77% and 23% at Banchare‐Sisdole, and 67% and 33% at Banchare. During the monsoon, fiber proportions increased at Sisdole and Banchare‐Sisdole compared to the premonsoon period, whereas a decrease was observed at Banchare (Figure 3). For comparison, Table 4 summarizes the morphology of MPs in soil reported in previous studies.

The elevated fiber concentration at Sisdole Upstream during the monsoon may be attributed to agricultural runoff from upstream areas, where fibrous MPs deposited via atmospheric pathways are likely mobilized and transported into the river. Previous studies have reported substantial MPs contamination in agricultural runoff [50–53], supporting this pathway. A further increase in fiber abundance at Sisdole Downstream is likely driven by landfill runoff, where fiber‐rich soils are mobilized during rainfall and enter the river system. In contrast, the reduction in fiber concentration at Banchare Downstream may be explained by dilution effects associated with tributary inflow, which increases river discharge.

Microbial degradation becomes more dominant than photodegradation in deeper soil layers, where sunlight penetration is limited [54]. Microorganisms can secrete enzymes that depolymerize MPs into short‐chain molecules, which may then be utilized for carbon and energy mineralization [55]. However, soil microbes lack the necessary enzymes to effectively break down synthetic polymers [56]. Therefore, as biodegradation requires prior depolymerization of MPs into small monomers before microbial utilization [57], the biodegradation of MPs in landfills is limited.

A previous study conducted at the Sisdole LFS by Bijay and Kumar [58] identified a relatively high proportion of textiles (5.28%) in MSW, which was notable compared to plastics (12%), one of the major components of MSW. Similarly, Khadka et al. [59] reported higher fractions of textiles (13%) and plastics (19%) in household MSW within Kathmandu Metropolitan City. Textiles continuously release fibers throughout their lifecycle due to mechanical, thermal, chemical, and biological degradation [60–62]. Hence, the dominance of fibrous MPs may be attributed to fiber emissions from textile waste, with rainfall‐induced leaching further enhancing their concentrations during the monsoon.

Given the detection limit of 90 μm, all analyzed particles were sufficiently large for reliable visual identification, suggesting that the observed fiber dominance reflects actual environmental patterns rather than methodological bias. In contrast, fragments, which are typically smaller, may be present in substantial quantities below the 90 μm detection threshold.

### 3.3. Size and Color of MPs

**FIGURE 4:**
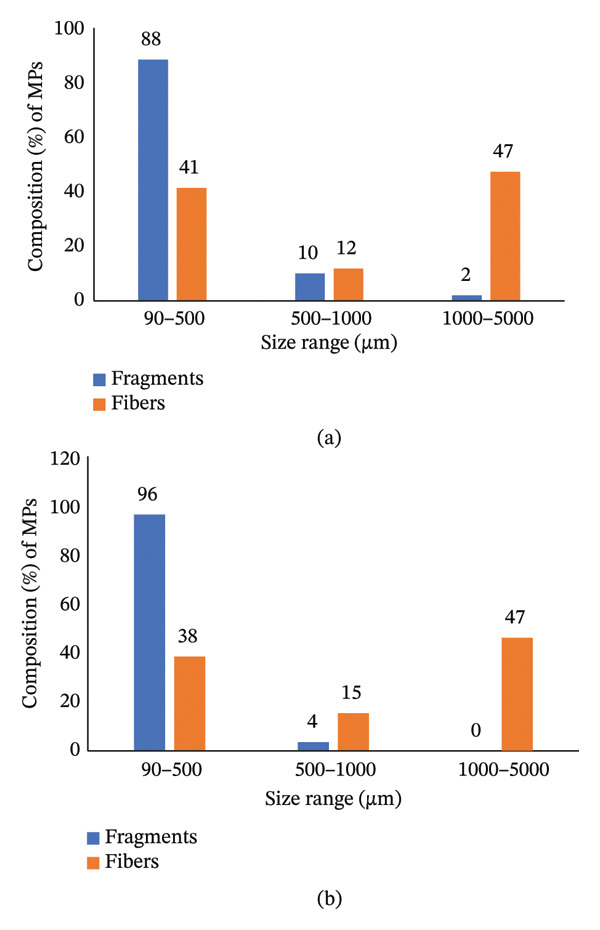
Size distribution of fibers and fragments observed in (a) leachate and (b) river water.

A large proportion (77%) of MPs in leachate were detected within the 90–500 μm size range, whereas MPs measuring 500–1000 μm and 1000–5000 μm represented 10% and 13% of the total MPs, respectively. The size distribution analysis revealed that fibers were frequently present in the 90–500 μm and 1000–5000 μm categories, while fragments were predominantly concentrated in the 90–500 μm range (Figure 4). Similarly, in water samples, 69% of the total MPs were within the 90–500 μm range, followed by 9% in 500–1000 μm and 22% in 1000–5000 μm. Consistent with the leachate findings, fragments were most abundant in the 90–500 μm range, whereas fibers were commonly observed in both the 90–500 μm and 1000–5000 μm ranges (Figure 4). For comparison, Table 3 summarizes the dominant MPs size ranges reported in previous leachate studies.

Smaller MPs (90–500 μm) accounted for 69% of the total MPs in soil samples, followed by 13% in 500–1000 μm and 18% in 1000–5000 μm. Fragments were predominantly found in 90–500 μm (93%), with 6% in 500–1000 μm and 1% in 1000–5000 μm. In contrast, fibers were more abundant in larger size categories, with 49% in 1000–5000 μm, 27% in 500–1000 μm, and 24% in 90–500 μm. Table 4 summarizes the dominant MPs size distribution in soil reported in previous studies for comparison with the present findings.

The dominance of fragments in smaller size classes can be attributed to their origin. Landfills, although generally well enclosed, expose plastics to harsh conditions such as fluctuating temperature, variable pH, high salinity, microbial activity, and physical stress, which promote fragmentation [13]. These processes lead to progressive disintegration, generating numerous tiny particles [63]. In contrast, fibers primarily originate from textiles and plastic ropes; due to their elongated morphology, they are more frequently observed in larger size classes.

**FIGURE 5:**
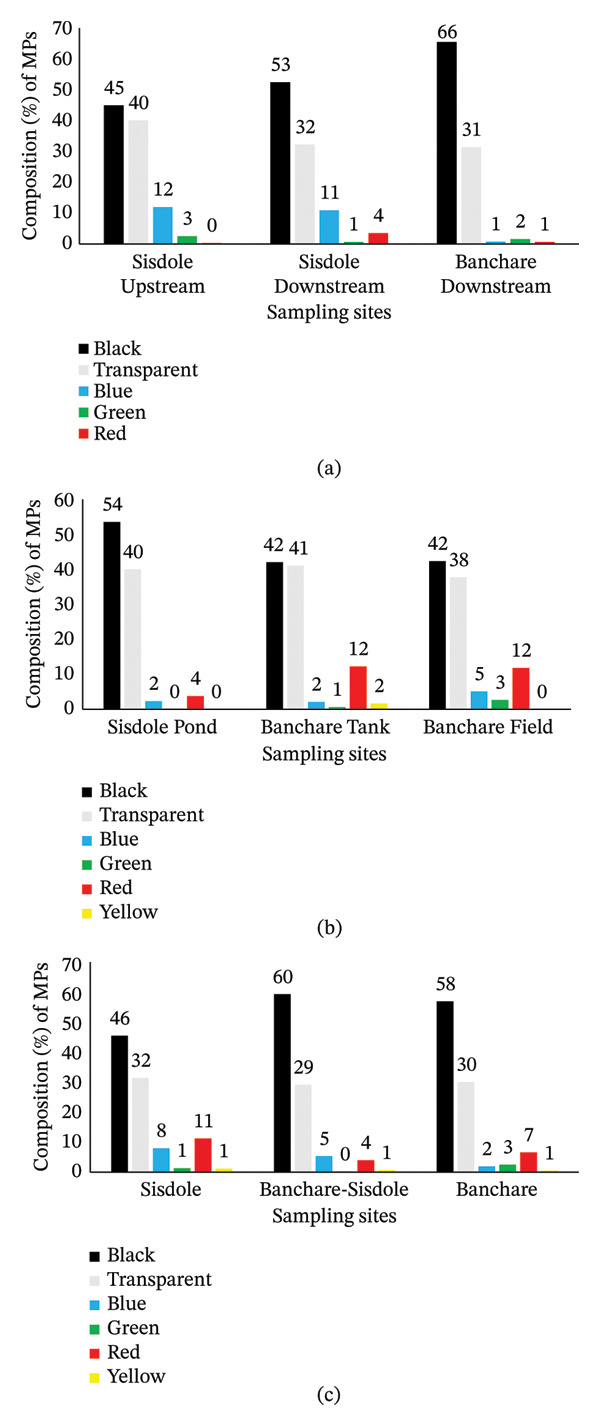
Composition of MPs in (a) river water, (b) leachate, and (c) soil.

Six different colors of MPs were identified in leachate, river water, and soil samples. In both leachate and river water, black (51%) was the most prevalent, followed by transparent ones (37%). Blue, red, and green contributed 6%, 5%, and 1%, respectively. Similarly, in soil samples, black MPs dominated (55%), while transparent MPs comprised 31%. The remaining colors: red, blue, green, and yellow, represented 7%, 5%, 1%, and 1%, respectively. Overall, black and transparent were the dominant colors across all sites (Figure 5).

Black and transparent particles have been reported as the predominant colors in landfill leachate and soil, along with blue, red, yellow, and other colors in previous studies [28, 29, 35, 37, 64]. The dominance of black and transparent MPs is likely associated with the widespread use and fragmentation of similarly colored plastic packaging materials and containers observed at the LFSs. Moreover, the similarity between commonly used textile colors and the predominance of fibrous MPs further supports textiles as an important source of fiber contamination in the study area.

### 3.4. Polymer Type

**FIGURE 6:**
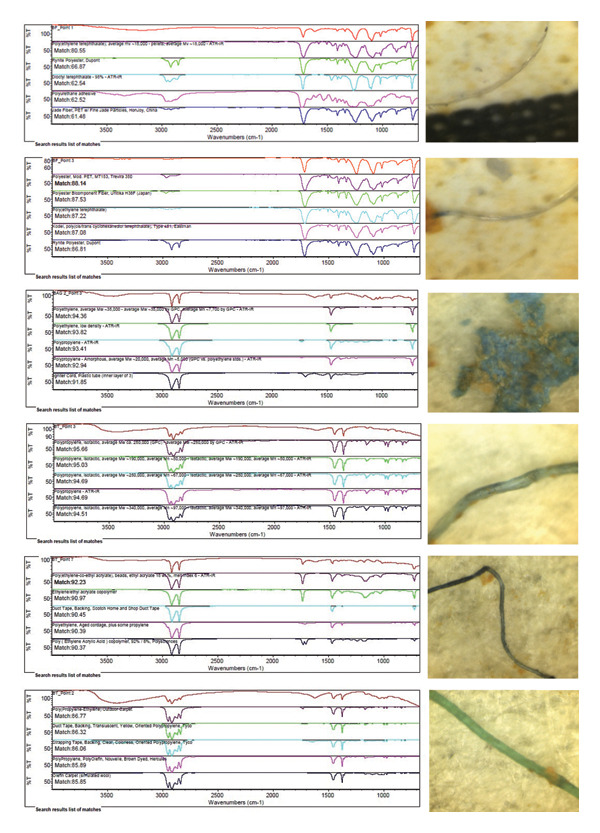
Image of MPs with μ‐FTIR spectra.

The polymer analysis identified six types of synthetic polymers (Figure 6). Among them, PES/PET was the most dominant (50%), followed by PP (20%) and PE (10%). Additionally, polymers such as poly(PP‐PE) and poly(ethylene‐co‐ethyl acrylate) together accounted for 20% of the total MPs. Fibers were mainly composed of PET, PES, poly(PP‐PE), and poly(ethylene‐co‐ethyl acrylate), whereas PP and PE were observed as fragments. The polymeric composition of MPs in leachate and soil reported in previous studies is summarized in Tables 3 and 4, respectively, for comparison with the present findings.

PET/PES fibers are widely recognized as key components of clothing and other textile products [65, 66]. The predominance of these polymers supports the previously suggested interpretation that fibers originate mainly from textiles and related materials. In contrast, PE is widely employed in the manufacture of plastic bottles, bags, and food containers [67], while PP is a common thermoplastic resin found in packaging bags and plastic films [68]. The considerable abundance of PP and PE MPs is consistent with the previously suggested interpretation that these fragments originated from plastic packaging and containers. The dominance of PET/PES, PP, and PE in LFSs can be attributed to their widespread use in both domestic and industrial waste streams in Nepal.

Although not included in the quantitative enumeration, natural/modified polymers, such as cellulose, rayon, and linen, were observed in notable proportions during polymeric analysis, accounting for 73%, 18%, and 9%, respectively. These fibers can also adsorb chemical pollutants and, due to their faster degradation, release them more readily into the surrounding environment than slowly degrading synthetic MPs [69]. They may also reach nearby agricultural lands via atmospheric deposition or the irrigation of leachate‐contaminated river water, potentially affecting soil quality and crops. However, these fibers were excluded from the quantitative enumeration because the study focused on synthetic polymers, which are more persistent and environmentally relevant. Synthetic polymers, due to their persistence, bioaccumulative potential, and capacity to adsorb hazardous contaminants, pose a substantial risk to ecological integrity and human health [70].

### 3.5. Implications for Exposure and Risk

The prevalence of MPs in river water (Banchare Downstream) and agricultural land (Banchare‐Sisdole) suggests potential risks to soil health and ecosystem functioning. As river water downstream from landfills is used for irrigation, MPs can be transferred from water to agricultural soils. Therefore, soil and water contamination represent a primary exposure pathway for MPs originating from the Banchare Dada and Sisdole LFSs.

The presence of MPs can affect soil health and fertility by altering soil pH, porosity, water‐holding capacity, and microbial enzymatic activities [71]. PET with phenyl groups has been found to exert the strongest effects on plant fresh weight, chlorophyll (a and b), and water content among different MPs types [72], and the dominance of PET/PES (50%) in this study suggests potential biological and physiological implications for agricultural systems. Bakhshaee et al. [73] further reported that HDPE MPs influence soil water holding capacity (WHC), with fragments increasing WHC more than fibers (36.3% vs. 19.8%). PE MPs have been shown to reduce plant growth and nitrogen uptake by inhibiting chlorophyll synthesis, suppressing nitrification processes, and decreasing ammonia‐oxidizing microbial activity, while also inducing oxidative stress in plants [74]. They affect soil‐plant systems by disrupting nutrient dynamics and soil fertility, impairing growth performance through reduced biomass, leaf development, and reproductive output, and altering root morphology, biomass allocation, soil structure, and microbial activity [75]. Overall, the dominance of PET/PES and the presence of PE in the studied samples suggest that MPs may adversely affect soil physical, chemical, and biological functioning by altering water retention, nutrient cycling, and soil fertility, while also impairing plant health through oxidative stress, reduced chlorophyll synthesis and nutrient uptake, and diminished growth, biomass, and reproductive performance.

However, the toxicity of MPs to soil and plant health depends on both dose and particle size. MPs in the size range of 300–600, 600–2000, and 2000–5000 μm showed size‐dependent effects on soil health indicators, with the smallest fraction (300–600 μm) causing the greatest disruption to soil nutrient retention and organic matter dynamics [76]. Similarly, Fang et al. [77] applied MPs in the size range of < 35, < 125, and < 500 μm at concentrations of 0, 0.1, 1.0, and 10 wt% and found that the smallest particles (< 35 μm), particularly at the highest concentration (10 wt%), caused the greatest reduction in mean weight diameter and soil aggregate stability, indicating stronger impacts of smaller and higher‐dose MPs on soil structure. In a study by Xu et al. [78], MPs in the size range of 1000–2000, 200–1000, 50–200, and 1–50 μm showed clear size‐dependent toxicity in plants under equal mass exposure, with the smallest fraction (1–50 μm) causing the most severe growth inhibition, oxidative stress, and root damage due to higher uptake and mobility, indicating greater bioavailability and potential underestimation of risk based on mass alone. Studies show that increasing MPs’ dose and decreasing particle size intensify inhibitory effects, especially on root activity and biomass, whereas larger MPs mainly cause physical damage to plant roots [79]. Nevertheless, the effects of MPs on plants are species‐dependent, with different plant species showing varying levels and types of responses to exposure [80].

PE has been reported in all organs of grass carp, indicating its ingestion and distribution within aquatic organisms [81]. Exposure of Daphnia magna to small‐sized fragmented PE (17–34 μm) resulted in higher ingestion and greater toxic effects than MPs beads (≈40 μm), significantly reducing survival, feeding activity, body size, and reproduction [82]. In this study, the presence of PE in river water suggests a potential exposure pathway for aquatic fauna and potential transfer of MPs through the aquatic food web. However, the shape and size of MPs influence their exposure pathway and toxicity to aquatic organisms. In a study by Ding et al. [80], MPs toxicity was found to depend on particle size and aquatic species. The study employed MPs of different sizes (0.03, 0.5, 5, and 20 μm), revealing size‐dependent toxicity with the highest effects at 0.03 μm, as well as species‐dependent responses among different algal species.

Furthermore, MPs can act as vectors for various contaminants by adsorbing pollutants, such as persistent organic pollutants (POPs) and heavy metals, as well as bacteria and viruses [83]. A recent study confirmed that MPs in leachate act as carriers of heavy metals [84]. The toxicity of MPs is additionally affected by the presence of co‐occurring pollutants [80]. In this study area, elevated concentrations of heavy metals, including zinc, cadmium, iron, and copper, have been reported in river water and leachate, exceeding FAO irrigation water guidelines [85]. Metals, such as cadmium, can be adsorbed onto PET and PP polymers due to their surface properties and polarity [86]. Therefore, the presence of PET and PE MPs facilitates the transport and potential bioaccumulation of these toxic substances in soil and along the food web. Such contamination may pose risks to human and animal health via ingestion of contaminated produce, as reported in previous studies [87–89].

Moreover, studies have shown that MPs may exacerbate landfill odor emissions by promoting sulfate reduction [90], while PE MPs have also been reported to enhance hydrogen sulfide (H_2_S) production during anaerobic treatment [91]. These findings suggest that the occurrence of MPs in the studied landfill system may contribute to odor formation through similar anaerobic pathways.

## 4. Limitations

This study was conducted with the following limitations:

• Due to methodological constraints, only MPs larger than 90 μm were analyzed. This may lead to an underestimation of total abundance, particularly of smaller, more mobile, and bioavailable particles that could pose higher ecological and health risks through ingestion and translocation. Furthermore, this limitation reduces the relevance for toxicological assessment.
• The polymer composition identified from the combined analysis of leachate and water samples was assumed to reflect soil contamination characteristics. However, other sources, such as agricultural plastics, surface runoff, or background atmospheric MPs, may also contribute to and influence the observed polymer profile.
• Polymeric characterization could not be performed separately for both seasons due to financial constraints. Bulk samples representing both seasons were created, and only 10 particles per filter were analyzed, which may mask seasonal variations in polymer composition.
• Sampling was limited to two campaigns. A year‐long analysis covering all seasons would provide a more comprehensive understanding of temporal variations in MPs concentration in leachate and soil.
• The study did not examine the ecological or toxicological impacts of MPs on soil or river water, limiting understanding of potential environmental risks.

## 5. Conclusion and Recommendations

MPs were detected at all sampling sites and across all sample types, suggesting a possible association between landfill activities and MPs contamination in surrounding water bodies and agricultural soils. The elevated concentration of MPs in active landfills compared to older ones suggests that the potential for MPs release declines with landfill age. The lower MPs concentration in the leachate collection tank relative to the field samples implies that daily soil cover applied after waste placement may help retain MPs within the landfill. Furthermore, the detection of MPs in agricultural soils suggests that atmospheric deposition from the landfill may contaminate nearby areas.

The dominance of PET/PES and the occurrence of PE in the studied samples suggest that MPs may adversely affect soil physical, chemical, and biological functioning by altering water retention, nutrient cycling, and soil fertility, while also impairing plant health through oxidative stress, reduced chlorophyll synthesis and nutrient uptake, and diminished growth, biomass, and reproductive performance. The detection of MPs in river water indicates a potential exposure pathway for aquatic organisms and the transfer of MPs through the aquatic food web. In addition, PET and PE MPs may facilitate the transport and bioaccumulation of toxic substances in soil and across food webs, potentially posing risks to human and animal health through the ingestion of contaminated produce. Furthermore, the occurrence of MPs in the studied landfill system may contribute to odor formation through anaerobic degradation pathways. Overall, these findings highlight the importance of effective leachate management and appropriate protective measures to minimize MPs contamination and exposure in water and agricultural environments.

Based on these findings, several recommendations are proposed for both responsible authorities and future research.

For responsible authorities:

• Given the elevated MPs concentrations observed in Banchare Downstream and Banchare Tank, leachate should be properly treated using integrated membrane bioreactor + ultrafiltration + nanofiltration (MBR + UF + NF) processes before discharge to reduce MPs contamination and protect irrigation water quality. In a study by Kara et al. [94], MBR + UF achieved an MPs removal efficiency of approximately 96% from landfill leachate, while the addition of nanofiltration (MBR + UF + NF) increased the efficiency to about 99%. In another study, MBR + UF + NF units achieved an overall MPs removal efficiency of approximately 97% [43].
• To prevent runoff and soil contamination during the monsoon, barriers and vegetation cover should be established around the Sisdole LFS, where higher MPs loads were detected in surrounding soils and river water.
• Waste management strategies should prioritize diverting plastics away from landfills, especially at sites contributing to high MPs release. Promoting recycling, upcycling, and enforcing the polluter pays principle will help reduce plastic waste inputs and mitigate environmental contamination.

For future research:

• Further studies should investigate the effects of leachate‐contaminated water on aquatic organisms and agricultural productivity when used for irrigation.
• The impacts of MPs on soil quality and crop yield should be collectively assessed to better understand their long‐term environmental implications.
• The transport and fate of MPs in soil should be examined to improve understanding of their environmental behavior.

## Author Contributions

Sandhya Babel: conceptualization, writing–review and editing, resources, and corresponding author.

Yubraj Dahal: conceptualization, methodology, sample collection and analysis, acquisition and analysis of data, writing–original draft, review and editing, and corresponding author.

Saroj Babu Koirala: conceptualization, sample collection and analysis, acquisition and analysis of data, and writing–original draft.

Ramesh Gautam: conceptualization, sample collection and analysis, acquisition and analysis of data, and writing–original draft.

Rashika Pandit and Reema Lama Pakhrin: laboratory work, acquisition and analysis of data, and writing–original draft.

Rabindra Prasad Dhakal: resources.

## Funding

This research was supported by the Nepal Academy of Science and Technology, Nepal (Fiscal Year 2081–2082 BS), and the Thailand Science Research and Innovation Fundamental Fund, Thammasat University, Thailand (Fiscal Year 2026).

## Ethics Statement

The authors have nothing to report.

## Consent

The authors have nothing to report.

## Conflicts of Interest

The authors declare no conflicts of interest.

## Data Availability

Data will be made available upon a suitable request.
